# Psychiatrists' attitudes towards autonomy, best interests and compulsory treatment in anorexia nervosa: a questionnaire survey

**DOI:** 10.1186/1753-2000-2-40

**Published:** 2008-12-17

**Authors:** Jacinta OA Tan, Helen A Doll, Raymond Fitzpatrick, Anne Stewart, Tony Hope

**Affiliations:** 1 The Ethox Centre, Department of Public Health, University of Oxford, Oxford, UK; 2 Department of Public Health, University of Oxford, Oxford, UK; 3 Oxfordshire and Buckinghamshire Mental Health Foundation NHS Trust, Oxford, UK

## Abstract

**Background:**

The compulsory treatment of anorexia nervosa is a contentious issue. Research suggests that psychiatrists have a range of attitudes towards patients suffering from anorexia nervosa, and towards the use of compulsory treatment for the disorder.

**Methods:**

A postal self-completed attitudinal questionnaire was sent to senior psychiatrists in the United Kingdom who were mostly general adult psychiatrists, child and adolescent psychiatrists, or psychiatrists with an interest in eating disorders.

**Results:**

Respondents generally supported a role for compulsory measures under mental health legislation in the treatment of patients with anorexia nervosa. Compared to 'mild' anorexia nervosa, respondents generally were less likely to feel that patients with 'severe' anorexia nervosa were intentionally engaging in weight loss behaviours, were able to control their behaviours, wanted to get better, or were able to reason properly. However, eating disorder specialists were less likely than other psychiatrists to think that patients with 'mild' anorexia nervosa were choosing to engage in their behaviours or able to control their behaviours. Child and adolescent psychiatrists were more likely to have a positive view of the use of parental consent and compulsory treatment for an adolescent with anorexia nervosa. Three factors emerged from factor analysis of the responses named: 'Support for the powers of the Mental Health Act to protect from harm'; 'Primacy of best interests'; and 'Autonomy viewed as being preserved in anorexia nervosa'. Different scores on these factor scales were given in terms of type of specialist and gender.

**Conclusion:**

In general, senior psychiatrists tend to support the use of compulsory treatment to protect the health of patients at risk and also to protect the welfare of patients in their best interests. In particular, eating disorder specialists tend to support the compulsory treatment of patients with anorexia nervosa independently of views about their decision-making capacity, while child and adolescent psychiatrists tend to support the treatment of patients with anorexia nervosa in their best interests where decision-making is impaired.

## Background

Patients suffering from anorexia nervosa may refuse treatment. One of the ethical issues pertinent to the management of treatment refusal is that of competence, or the ability of patients to make their own treatment decisions. It is generally agreed that patients who possess the competence to make treatment decisions should be allowed to make their own treatment choices, even if these choices appear to be foolish or unwise [1,2]. The legal criteria of this ability of competence in the United Kingdom, which is called capacity, generally focus on abilities to understand, retain and weigh treatment information, to come to a decision, and to express a choice (see the Mental Capacity Act 2005 and Adults with Incapacity (Scotland) Act 2000). Research suggests that there can be additional areas in which patients with mental disorder can have difficulties with making decisions, such as appreciation (applying information to oneself) [3]. Furthermore, research suggests that for anorexia nervosa in particular, patients can experience difficulties with making decisions to accept treatment because of shifts in value systems, the incorporation of the mental disorder in the patient's sense of personal identity, and battles for control with mental health professionals [4-7]. As anorexia nervosa is a relatively rare mental disorder, most general psychiatrists treat relatively few patients with anorexia nervosa and may not feel highly skilled in its management. At the same time, the paucity and uneven distribution of dedicated eating disorder services [8,9] means that it is likely that the majority of patients with anorexia nervosa in the United Kingdom would be seen and treated by general psychiatrists who do not have special expertise in treating eating disorders.

There is relatively little known about the frequency of use of compulsory treatment in anorexia nervosa. Legislation relevant to compulsory treatment of anorexia nervosa in legal minors and adults varies internationally [10]. In England and Wales, the Mental Health Act 1983 (now amended by the Mental Health Act 2007) allows compulsory treatment of mental disorders across all ages, so long as there is risk to the person or others. In Scotland, the Mental Health (Care and Treatment) (Scotland) Act 2003 also allows compulsory treatment of mental disorders in the presence of risk to health or safety, so long as the mental disorder is impairing the ability of the patient to make treatment decisions. Mental health professionals in England and Wales may use the Children Act 2004 to provide care and treat legal minors (those under the age of 18 years) without consent in the interests of their welfare. Legal minors may also be treated without their consent if parental consent is given. In the United Kingdom, a survey by the Royal College of Psychiatrists in 1992 found that 9% of inpatient anorexia nervosa patients in the United Kingdom were given compulsory treatment under the Mental Health Act 1983 [8]. An English specialist adult eating disorder centre receiving nationwide referrals of particularly difficult cases reported a rate of use as high as 16% [11]. An American specialist eating disorder unit also reported a similar rate of 16.6% (66 out of 397 inpatient admissions) compulsory inpatient admissions over a period of 7 years [12].

With regard to the course and outcome of compulsory treatment in anorexia nervosa, there are only a small number of studies. Some studies have looked at the legal pathways to implementation of compulsory treatment in anorexia nervosa [13,14] and others have examined the effectiveness of inpatient hospitalisation or compulsory treatment in anorexia nervosa [11,12,14,15]. Two studies found no difference between compulsory treatment and voluntary inpatient admissions on outcomes such as weight restoration [11,12] but one of these found a poorer outcome in terms of mortality at 5 years [11]. However, randomised trials of compulsion have not been possible and there is evidence that compulsorily treated patients may differ from patients of similar severity and duration of illness who are treated voluntarily. Compulsorily treated patients tend to have a greater number of previous admissions, a history of childhood sexual or physical abuse or previous self-harm, and a lower WAIS-R full IQ score, which may point to a more intractable nature of disorder or a lower competence to make treatment decisions [11,12].

In terms of research on this topic to date there have been a few studies of the views and attitudes of psychiatrists or other mental health professionals towards patients with anorexia nervosa. These studies have looked at their understanding of the disorder [16] and their attitudes to patients with their disorder [17,18]. One questionnaire survey found that patients with eating disorders were less liked than patients with schizophrenia and were seen as responsible for their illness almost to the same degree as people who take recurrent overdoses. Factor analysis showed a factor in which patients with eating disorders were construed as vulnerable to external pressures (such as from others and the media) and, moreover, their illness was seen as self-induced. This was associated with agreement with treatment recommendations for education, which urge the patient to take control and accept psychotherapy [17].

There have been some empirical studies exploring psychiatrists' attitudes towards the implementation of compulsory treatment in general [19-23], as well as the pattern of use of mental health legislation [24], but none focussing on anorexia nervosa in particular. One questionnaire survey found that psychiatrists' responses were influenced not only by the severity of and risks associated with the patient's disorder, but also by family pressure which affected the decision-making process [25]. No study has examined professionals' opinions of the disorder's impact on a patient's competence to make treatment decisions; views of the patients' treatment refusals; and views of the use of compulsory treatment in anorexia nervosa. Whether and to what extent these opinions are associated with characteristics of the clinicians have also not been previously studied.

## Methods

### Aims of the Questionnaire Study

This questionnaire survey, which was restricted to senior psychiatrists, had four aims:

1. To determine the range of attitudes amongst psychiatrists towards competence to make treatment decisions and treatment refusal by patients with anorexia nervosa.

2. To explore the factors that psychiatrists regard as relevant to the consideration of the use of compulsory treatment in anorexia nervosa, and to determine which factors they consider particularly important.

3. To explore how psychiatrists use concepts such as competence, and the patients' recognition of their own best interests, in their decisions.

4. To examine the relationships between psychiatrists' characteristics and their views about these issues.

### Development and implementation of the questionnaire survey

A self-administered postal attitudinal questionnaire was piloted on a small number of ethicist and clinician volunteers. The questionnaire format and items were developed through an iterative process of clarifying and simplifying the questionnaire [26]. The final questionnaire contained 37 attitudinal items, each item having 7 possible responses (scored 0 to 6) on an ordinal scale.

In October 2004, the questionnaires, which also asked about respondents' duration of practice as a psychiatrist, psychiatric speciality, age and sex ('respondent characteristics'), were mailed out. Questionnaires were sent to all psychiatrists who had consented to receive research mailings, and who belonged to the South East Region General Adult Faculty, the South East Region Child and Adolescent Faculty, or the Eating Disorder Special Interest Group (EDSIG) of the Royal College of Psychiatrists across the United Kingdom. Eating Disorder Special Interest Group membership was open to any members of the Royal College interested in eating disorders. In 2007, after the study was conducted, the Eating Disorder Special Interest Group became the Eating Disorders Section of the Royal College. A second set of mailings was sent to non-responders in February 2005. Returns were received from February 2005 until the end of March 2006.

### Statistical methods

Responses to the questionnaire and respondent characteristics items are reported throughout as 'N (valid %)'. (Valid percentages are percentages calculated using only the number of people who answered a particular question.) Chi-squared and Wilcoxon matched pairs signed ranks tests were used to determine the statistical significance of associations between respondent characteristics and attitudinal item scores, and to examine the associations between individual questionnaire items, respectively.

To assess whether there were certain consistent patterns of response across questionnaire items, and thus to identify the presence of specific underlying factor(s), an exploratory principal components factor analysis (using both Varimax and direct Oblimin rotations) was performed on all 37 questionnaire items. Factors were selected if their Eigenvalues were at least 1.0, or if the Eigenvalues occurred before the slope of the scree plot slope began to plateau. The items contributing to each factor were taken as those with a factor loading of at least 0.40. A total factor score was obtained by summing, after correcting for directionality, the item responses for the component items. Each total factor score was standardised, for ease of comparison, by dividing by the maximum possible score and multiplying by 10, so as to obtain a number between 0 and 10. A higher score indicates a greater tendency to provide responses to questionnaire items that contribute to the particular construct. Cronbach's alpha was used to assess the internal reliability of each factor, with an alpha of 0.70 or more indicating internal reliability. Convergent and construct validity were assessed by cross-correlating the individual factor scores using Spearman's correlation coefficient.

Factor scores were compared individually between groups of respondents (males versus females; eating disorder specialists versus non-eating disorder specialists; EDSIG members versus non-EDSIG members; those with up to 10 years of practice versus those with more than 10 years of practice; and child and adolescent psychiatrists versus non-child and adolescent psychiatrists). Scores were compared between each individual category using both t-tests and multiple linear regression analysis, the latter to adjust for other respondent characteristics.

## Results

### Response rate and respondent characteristics

Of the 1482 names on the mailing list, 160 individuals were excluded as they no longer lived at the addresses given on the lists, or they had left the country. Of the remaining 1322 individuals, 686 returned a completed questionnaire, giving a response rate of 51.9%.

Slightly more than half of the respondents were male (n = 357, 52.2%). The majority (n = 496, 72.4%) were consultant psychiatrists, with 440 (64.2%) having worked as psychiatrists for more than 10 years. Almost all of the respondents (n = 598, 87.5%) held Mental Health Act approval status, which requires training in the implementation of mental health legislation.

209 (30.4%) of the respondents said they were child and adolescent psychiatrists and 366 (56.4%) that they were general adult psychiatrists. 139 (20.3%) respondents belonged to other subspecialties such as psychotherapy and forensic psychiatry.

With respect to eating disorders, 39 (5.7%) respondents worked in settings where they only treated eating disorder patients. A further 68 (10.0%) of respondents said that they worked in settings with special interest in eating disorders. When asked what they considered their specialty area(s), 62 (9.1%) of respondents reported that they were eating disorder specialists. All these individuals comprised a total of 108 (15.7%) classified overall in this analysis as eating disorder specialists. Note that the number of EDSIG members who responded to the questionnaire was 245 and that therefore fewer than half of EDSIG members are categorised as eating disorder specialists. Note also that individuals could be counted in more than one category.

The respondents saw patients with a spread of ages, with 534 (78.0%) of the respondents seeing patients in the age range of 13 to 25 years covered by the legal and ethical issues examined in this study and in the qualitative studies already conducted.

Respondents were asked how many patients with anorexia nervosa they had seen in the previous twelve months who had: outpatient treatment; day patient treatment; inpatient treatment (on a voluntary basis) or inpatient treatment (compulsorily treated at any time during admission). The results are shown in Table 1.

**Table 1:** Distribution of questionnaire respondents according to number of patients seen in each treatment setting in the previous 12 months.

**Treatment setting**	**Respondents distributed according to the number of patients with anorexia nervosa they had seen in each type of treatment setting in the previous 12 months (valid percentage of all respondents)**				
	**No patients**	**1 – 10 patients**	**11 – 20 patients**	**21 – 30 patients**	**> 30 patients**

**Outpatient**	25.3%	63.4%	5.5%	2.2%	3.6%

**Day patient**	79.2%	17.9%	1.5%	0.8%	0.5%

**Inpatient, voluntary status throughout admission**	59.4%	33.8%	4.0%	1.8%	1.0%

**Inpatient, on Mental Health Act at some point in admission**	76.6%	22.9%	0.3%	0.2%	0

Counting individual patients seen in more than one setting in each and every applicable setting (valid percentages of respondents)

The majority of respondents had seen between 1 and 10 patients with anorexia nervosa in an outpatient setting in the previous 12 months, with 17.9% having seen between 1 to 10 patients in a day patient setting. The majority of respondents had not seen any inpatients with anorexia nervosa, but it is interesting to note that over one fifth of the respondents had looked after patients with anorexia nervosa in a compulsory inpatient setting, and over a third had looked after patients in an inpatient setting who had never been on compulsory treatment. Overall, therefore, a large proportion of the respondents had recent practical experience of looking after patients with anorexia nervosa in both outpatient and inpatient settings, but it was a very small minority who had extensive experience in this.

The relatively small number of respondents who saw any patients in the day patient setting probably reflects the small number of units across the United Kingdom that offer day patient facilities tailored for anorexia nervosa [9]. Day patient programmes for anorexia nervosa tend to require staff training and physical facilities that are different from those for other mental disorders, because of the emphasis on supervision of meals, prevention of excessive activity and monitoring of physical ill health caused by the disorder. These day patient programmes are mainly found in specialist eating disorder settings or special interest eating disorder settings. It should also be noted that not all specialist eating disorder units in the United Kingdom admit inpatients compulsorily.

### Questionnaire responses

The responses of the respondents to selected questionnaire items are summarized in Tables 2 to 4, in the order in which the results are described below by topic.

**Table 2:** Attitudes to the use of the Mental Health Act (Valid % responses)

Part B – Questions on mental disorders in general							
	Strongly disagree	Moderately disagree	Slightly disagree	Neither agree nor disagree	Slightly agree	Moderately agree	Strongly agree

B8. 'The Mental Health Act should be used more frequently to protect the health and safety of patients.'	9.1	15.3	13.4	35.6	11.3	10.9	4.4

B9. 'The Mental Health Act should not be used when patients are able to make informed treatment decisions, even if they are placing themselves at risk.'	6.3	18.7	16.3	7.4	15.0	24.6	11.6

B10. 'The Mental Health Act should not be used to enforce admission to hospital for mental disorders.'	73.7	18.4	3.2	1.2	0.9	1.3	1.3

B11. 'The Mental Health Act is used too often in the treatment of mental disorders.'	13.3	23.0	14.0	31.1	12.2	4.1	2.4

Part C – Questions specifically on anorexia nervosa:							

I. Use of the Mental Health Act for anorexia nervosa							

	Strongly disagree	Moderately disagree	Slightly disagree	Neither agree nor disagree	Slightly agree	Moderately agree	Strongly agree

C12. 'The Mental Health Act should not be used when patients clearly believe that the advantages of anorexia nervosa for them outweigh the disadvantages.'	34.2	39.4	11.5	8.3	3.2	2.8	0.6

C13. 'It is appropriate that the Mental Health Act enables compulsory re-feeding of patients with anorexia nervosa.'	1.3	3.2	1.9	3.1	11.5	42.6	36.3

C14. 'The Mental Health Act is used too often in the treatment of anorexia nervosa.'	9.1	20.6	14.0	51.0	3.4	1.3	0.6

C15. 'The Mental Health Act should not be used to enforce admission to hospital for anorexia nervosa.'	42.6	38.2	10.6	4.3	1.9	1.9	0.4

C16. 'The Mental Health Act should be used more frequently to protect the health and safety of patients with anorexia nervosa.'	2.1	8.0	9.3	42.9	14.7	18.3	4.9

IV. The use of the Mental Health Act in anorexia nervosa							

'Imagine that you are treating a 19-year old female patient who has anorexia nervosa. She is not able to put on weight in the outpatient treatment setting but is refusing day or inpatient treatment. Each statement below is your clinical judgement of her current situation. Please decide the relative importance of each factor below with respect to the decision your clinical team should make about whether or not to place this patient on a Mental Health Act Section 3.'							

	Not important ↔ Very important						

Importance Score	**1**	**2**	**3**	**4**	**5**	**6**	**7**

E32. 'The patient's physical health is at risk.'	0.1	0.9	1.0	2.7	19.0	37.0	39.3

E33. 'The patient would die if not given treatment.'	0.1	0.4	0.4	1.2	2.6	14.5	80.6

E34. 'The patient is unable to recognise what is in her own best interests.'	1.2	2.8	3.8	11.9	23.4	31.2	25.7

E35. 'The patient is not making choices consistent with her pre-morbid personality or wishes.'	3.1	5.1	8.4	14.0	27.2	22.9	19.4

E36. 'The patient's family is unable to support her in the treatment.'	6.5	12.8	14.0	21.3	22.8	15.0	7.8

E37. 'The patient's family is keen to support use of compulsory treatment.'	11.3	18.4	17.4	22.9	19.4	7.4	3.2

**Table 3:** Views of the impact of having anorexia nervosa on competence (Valid % responses)

Part A – Vignette							
	Strongly disagree	Moderately disagree	Slightly disagree	Neither agree nor disagree	Slightly agree	Moderately agree	Strongly agree

A5. 'Although Mandy is intellectually able to understand the risks, the fact that she has anorexia nervosa means that her competence to refuse treatment is almost certainly compromised.'	5.7	10.3	7.2	4.6	16.2	32.8	23.2

Part C – Questions specifically on anorexia nervosa: II. Treatment decisions in anorexia nervosa							

	Strongly disagree	Moderately disagree	Slightly disagree	Neither agree nor disagree	Slightly agree	Moderately agree	Strongly agree

C20. 'Treatment refusal by patients is due to the influence of the anorexia nervosa and therefore does not fully reflect their true wishes or personality.'	1.5	7.5	11.3	8.0	24.3	36.8	10.6

Par A: 'Mandy is 16 years old, and is being treated in the community for anorexia nervosa. She is reluctant to put on weight as she feels she is too fat. She understands, at least intellectually, that if she continues to lose weight she can put her health and life at risk. Despite outpatient psychological treatment together with dietary advice, she continues to lose weight, and weighs 75% of her expected weight with associated physical symptoms. Medical investigations suggest her situation is medically serious but not yet life-threatening. She is resistant to the doctor's recommendation to be admitted to hospital. Her parents feel they cannot look after her at home any longer and want her admitted to hospital.'

**Table 4:** Attitudes to the impact of different severities of anorexia nervosa (Valid % responses)

Part C – Questions specifically on anorexia nervosa: III. Choice & responsibility in anorexia nervosa							
	Strongly disagree	Moderately disagree	Slightly disagree	Neither agree nor disagree	Slightly agree	Moderately agree	Strongly agree

'Patients with anorexia nervosa choose to engage in weight loss behaviours'							

D22. '- mild anorexia nervosa'	3.7	11.9	8.2	7.5	25.2	32.1	11.3

D23. '- severe anorexia nervosa'	20.2	28.4	9.9	6.9	11.4	13.0	10.2

'Patients with anorexia nervosa are able to control their own dieting, exercise and purging behaviours'							

D24. '- mild anorexia nervosa'	5.6	17.6	15.1	5.9	29.8	21.7	4.3

D25. '- severe anorexia nervosa'	34.1	35.7	9.4	5.5	8.6	3.7	3.1

'Patients with anorexia nervosa want help even when they are refusing it'							

D26. '- mild anorexia nervosa'	5.9	14.4	16.3	26.2	18.2	16.9	2.1

D27. '- severe anorexia nervosa'	9.8	16.7	10.2	27.4	14.2	17.8	4.0

'Patients with anorexia nervosa are generally able to reason properly about treatment'							

D28. '- mild anorexia nervosa'	5.2	22.0	23.3	10.0	21.5	15.8	2.2

D29. '- severe anorexia nervosa'	40.4	35.5	10.8	5.0	4.7	3.1	0.4

'Patients with anorexia nervosa have difficulties other than problems with reasoning that make it hard for them to make treatment decisions'							

D30. '- mild anorexia nervosa'	1.2	4.0	4.0	12.5	29.6	35.8	12.8

D31. '- severe anorexia nervosa'	1.5	2.4	1.6	8.0	11.6	39.3	35.7

#### (i) Attitudes to the use of the Mental Health Act (Table 2)

Approximately one third of respondents thought that the Mental Health Act is used appropriately to protect the health and safety of patients with mental disorders in general (item B8; 36%), and is not applied too often (item B11; 31%). While the support for the use of the Mental Health Act to enforce inpatient admission was high for anorexia nervosa in particular (item C16, 43%; item C14, 51%; both p < 0.001), it was significantly greater for mental disorders in general than for anorexia nervosa in particular, with 74% strongly disagreeing that the Mental Health Act *not *be used to enforce admission for mental disorders (item B10), compared with 43% for anorexia nervosa (item C15) (Wilcoxon z = -11.2; p < 0.001). There was, in addition, strong consensus for the use of the Act to enable compulsory re-feeding in anorexia nervosa, with 90.4% of respondents agreeing with this statement (item C13). In terms of factors considered relevant for the use of the Mental Health Act (Part C: IV in Table 2), respondents rated the risk of death as most important (item E33; 80% gave this the highest rating), followed by risk to physical health (item E32; 39%) with the inability of the family to support treatment (item E36) and the family being keen to support compulsory treatment (item E37) being rated the least important (8% and 3% of respondents giving each item the highest rating respectively).

#### (ii) The impact of having anorexia nervosa on competence (Table 3)

More than half (56%) of the respondents moderately or strongly agreed that anorexia nervosa compromises the competence of an adolescent to make treatment decisions (item A5). Almost three-quarters of respondents agreed with the statement that treatment refusal is due to the influence of the anorexia nervosa and does not fully reflect the patient's true wishes or personality (72% agreed, item C20).

#### (iii) Attitudes to the impact of different severities of anorexia nervosa (Table 4)

In order to allow for variations of perception of severity, the respondents were asked to interpret 'mild' or 'severe' anorexia nervosa as they normally would, rather than being provided with a set of criteria or clinical parameters. There were some clear, and significant, differences between respondents' attitudes to choice and responsibility in mild and severe anorexia nervosa. Generally, respondents agreed that patients with mild anorexia nervosa were choosing (69%) and able to control (56%) their behaviours (weight loss and dieting, exercise and purging – items D22 and D24 respectively), whereas respondents generally felt that patients with severe anorexia nervosa were *not *able to do so (59% and 79%, items D23 and D25, respectively). Similarly, while 40% of respondents believed that patients with mild anorexia nervosa were able to reason properly about treatment (item D28), only 8% of respondents believed this with regard to patients with severe anorexia nervosa (item D29). More respondents also strongly agreed that patients with severe anorexia nervosa had difficulty making treatment decisions (due to difficulties other than reasoning) (36%; item D31) than patients with mild anorexia nervosa (13%; item D30). Respondents in general considered that anorexia nervosa, whether mild or severe, makes it hard for patients to make treatment decisions. Respondents reported a similar and broad spread of opinions about whether patients with both mild and severe anorexia nervosa want help even when they are refusing it (items D26 and D27).

Psychiatrists who had expertise in the treatment of anorexia nervosa had some differences in their attitudes to 'mild' anorexia nervosa as compared to other respondents. Psychiatrists who classified themselves as eating disorder specialists or were working in eating disorder settings (who we will call eating disorder specialists) were strikingly less likely to think that patients with mild anorexia nervosa choose to engage in weight loss behaviours (item D22) (Chi-square = 10.80, d.f. = 2; p = 0.005). The eating disorder specialists had significantly different responses from other psychiatrists, being split almost equally between agreement and disagreement about whether patients with mild anorexia nervosa were able to control their dieting, exercise and purging behaviours, whereas other psychiatrists were more likely to think these patients were able to control these behaviours (item D24) (Chi-square = 6.184, d.f. = 2; p = 0.045). However, eating disorder specialists' responses were not significantly different from other psychiatrists in the items regarding whether or not patients with mild anorexia nervosa were able to want help, able to reason, and had difficulties with decision-making. (items D26, D28 and D30 respectively) (Chi-square = 1.98, d.f. = 2; p = 0.372; Chi-square = 3.373, d.f. = 2; p = 0.185 and Chi-square = 1.544, d.f. = 2; p = 0.462 respectively).

#### (iv) The views of child and adolescent psychiatrists compared to other respondents

As anorexia nervosa tends to occur in adolescents, the views of child and adolescent psychiatrists were of interest in this study. Statistical analysis was carried out for the responses of the child and adolescent psychiatrists, against those who did not classify themselves as child and adolescent psychiatrists. Analysis showed that child and adolescent psychiatrists had clearer opinions than the other psychiatrists on issues which were relevant only to the treatment of legal minors. Significant differences in responses were found for several items.

Child and adolescent psychiatrists were more likely to support the use of compulsory treatment under mental health legislation in the adolescent in the vignette (see Section A of the questionnaire), who was 16 years old (item A2) (Chi-square = 16.823, d.f. = 6; p = 0.010). Although there was a broad range of opinion amongst child and adolescent psychiatrists, most of them supported the use of parental consent, whereas non-child and adolescent psychiatrists did not (item A4) (Chi-square = 43.872, d.f. = 6; p < 0.001). The child and adolescent psychiatrists were much more inclined to agree that although a 16 year-old patient with anorexia nervosa is intellectually able to understand the risks, the fact that she has anorexia nervosa means that her competence to refuse treatment is almost certainly compromised (item A5) (Chi-square = 31.657, d.f. = 6; p < 0.001).

### Results of exploratory factor analysis

The exploratory factor analysis identified thirteen separate factors with Eigenvalues above the value of 1.0; these thirteen factors accounted for 64.6% of the total variance of the responses. The scree plot showed that the Eigenvalues of the first three factors occurred before the slope flattened. These three factors accounted for approximately a quarter (26%) of the variance, and had Eigenvalues of 2.0 and above. The remaining 9 factors individually contributed much less to the total variance. The Varimax and Oblimin rotations produced factors with the same items loading on each. The constituent questionnaire items for each factor (those loading 0.4 or more on each factor) and the distribution of the respondents' factor scale scores (standardised to a 0 to 10 scale) are shown in Table 5. The nature of the constituent items suggested that the following constructs underlie the factors:

**Table 5:** Emergent factors from the Exploratory Factor Analysis

Factor and its constituent questionnaire items	Cronbach's Alpha (test of internal reliability)
**Factor 1: Support for the powers of the Mental Health Act to protect from harm**	**0.70**
A7. Vignette item: 'If Mandy were 25 years old rather than 16 years old, her treatment team should be less willing to override her treatment refusal.' – reversed	
B10. 'The Mental Health Act should not be used to enforce admission to hospital for mental disorders.' – reversed	
B11. 'The Mental Health Act is used too often in the treatment of mental disorders.' – reversed	
C12. 'The Mental Health Act should not be used when patients clearly believe that the advantages of anorexia nervosa for them outweigh the disadvantages.'- reversed	
C13. 'It is appropriate that the Mental Health Act enables compulsory re-feeding of patients with anorexia nervosa.'	
C14. 'The Mental Health Act is used too often in the treatment of anorexia nervosa.' – reversed	
C15. 'The Mental Health Act should not be used to enforce admission to hospital for anorexia nervosa.' – reversed	
C16. 'The Mental Health Act should be used more frequently to protect the health and safety of patients with anorexia nervosa.'	
E32. Consideration of use of the Mental Health Act if: 'The patient's physical health is at risk.'	

**Factor 2: Primacy of best interests**	**0.75**
A2. 'Since the Mental Health Act permits compulsory treatment in this case, it should be used as she is at substantial risk.'	
A3. 'Since Mandy is young she should be treated in her best interests against her will.'	
A4. 'In the end the parents' decision should prevail over Mandy's treatment refusal as she is only 16 years old.'	
A5. 'Although Mandy is intellectually able to understand the risks, the fact that she has anorexia nervosa means that her competence to refuse treatment is almost certainly compromised.'	
C18. 'Treatment of anorexia nervosa against a patient's will is justified if it is likely that the patient will recover and have a good outcome after treatment.'	
C19. 'Treatment of anorexia nervosa against a patient's will is justified if it is likely that the patient will subsequently say he or she is glad that treatment was enforced.'	
C20. 'Treatment refusal by patients is due to the influence of the anorexia nervosa and therefore does not fully reflect their true wishes or personality.'	
Consideration of the use of the Mental Health Act if:	
E34. 'The patient is unable to recognise what is in her own best interests.'	
E35. 'The patient is not making choices consistent with her pre-morbid personality or wishes.'	
E36. 'The patient's family is unable to support her in the treatment.'	
E37. 'The patient's family is keen to support use of compulsory treatment.'	

**Factor 3: Autonomy viewed as being preserved in anorexia nervosa**	**0.71**
A1. 'Since Mandy understands the risks, her refusal of treatment should ultimately be respected.'	
A2. 'Since the Mental Health Act permits compulsory treatment in this case, it should be used as she is at substantial risk.' – reversed	
D22. 'Patients with anorexia nervosa choose to engage in weight loss behaviours – mild anorexia nervosa'	
D23. 'Patients with anorexia nervosa choose to engage in weight loss behaviours – severe anorexia nervosa'	
D24. 'Patients with anorexia nervosa are able to control their own dieting, exercise and purging behaviours – mild anorexia nervosa'	
D25. 'Patients with anorexia nervosa are able to control their own dieting, exercise and purging behaviours – severe anorexia nervosa'	
D28. 'Patients with anorexia nervosa are generally able to reason properly about treatment – mild anorexia nervosa'	

#### Factor 1: 'Support for the powers of the Mental Health Act to protect from harm'

**Figure 1:**
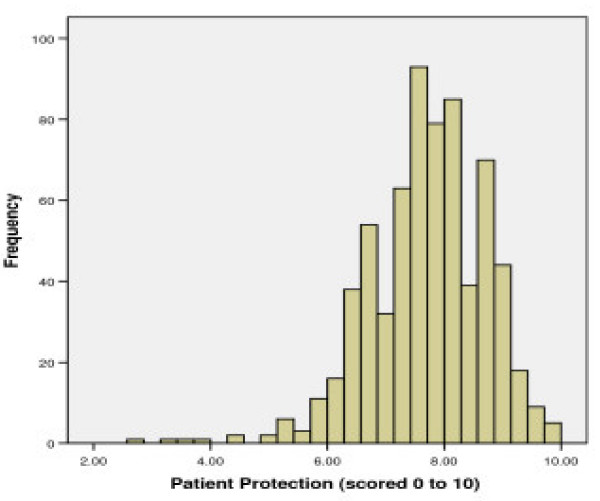
Distributions for scores on Factor 1: 'Support for the powers of the Mental Health Act to protect from harm'.

This factor includes 9 items which explained 13.7% of the variance in the item responses. It contains items describing the use of the Mental Health Act to protect people from the risk of harm, particularly harm to physical health. Higher scores on the factor reflect greater agreement with the principle of protection from harm. The distribution of standardised scores shows that the majority (95.8%) of respondents scored 6 or more, with the mean (SD) score being 7.69 (0.99) (See Figure 1).

#### Factor 2: 'Primacy of best interests'

**Figure 2:**
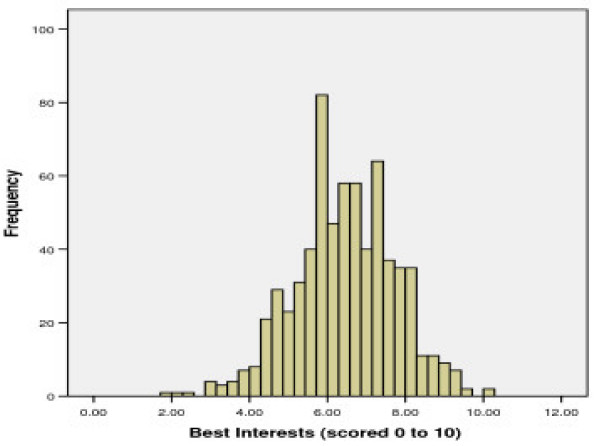
Distributions for scores on Factor 2: 'Primacy of best interests'.

This factor includes 11 items which explained 7.4% of the variance in the item responses. It contains items relating to the attitude that health professionals should act in the patient's best interests to protect patients who have anorexia nervosa because the anorexia can compromise and interfere with autonomy and decision-making. Higher scores reflect greater agreement with the use of the principle of best interests. The mean (SD) standardised score was slightly lower than for factor 1 at 6.43 (1.29), with the majority (96.9%) of respondents scoring 4 or more. (See Figure 2)

#### Factor 3: 'Autonomy viewed as being preserved in anorexia nervosa'

**Figure 3:**
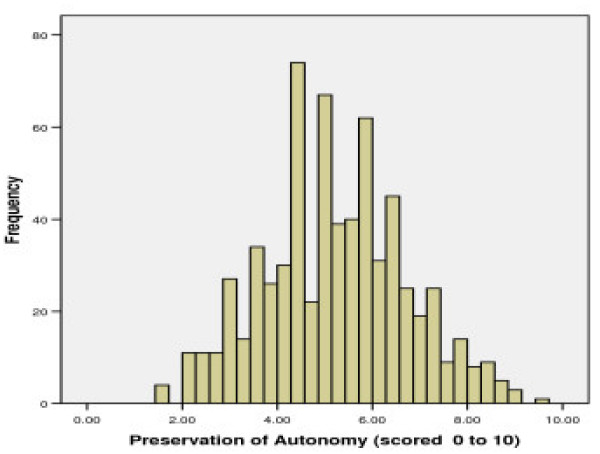
Distributions for scores on Factor 3: 'Autonomy viewed as being preserved in anorexia nervosa'.

This factor includes 7 items which explained 5.8% of the variance in the item responses. It contains items relating to the attitude that the nature of anorexia nervosa is such that it does not affect patients' choice, reasoning, control and responsibility for their own behaviours and decisions. Higher scores reflect greater agreement that autonomy is preserved in anorexia nervosa, with mean (SD) standardised score at 5.20 (1.50) being lower than those for factors 1 and 2, with the majority (99.7%) of subjects scoring 2 or more. (See Figure 3)

#### (i) Testing internal reliability of the factors

Each factor had a Cronbach's alpha of 0.7 or above, indicating that each has a high level of internal reliability (Table 5).

#### (ii) Relationships among the factors

There was a small to moderate but highly statistically significant positive correlation between respondents' responses on Factor 1 and Factor 2 (Spearman's rho 0.24, p < 0.001). There were also moderate and highly statistically significant negative correlations between Factors 1, 2 and 3 (Spearman's rho -0.35 and -0.36, respectively, both p < 0.001). These correlations support the construct validity of the factors, with both convergent and divergent validity being demonstrated in the expected directions. We would expect that individuals who espouse protection of patients from harm would also tend to espouse treatment in a patient's best interests, and that both attitudes would be negatively correlated with attitudes that the nature of anorexia nervosa does not lead to loss of autonomy and choice.

#### (iii) Relationships between the factors and categories of respondents

The relationships between the factors and categories of respondents are shown in Table 6. Since EDSIG membership was not significantly associated with scores on any of the three factors, even after adjustment for the other respondent characteristics, with the factor scores being similar in both groups, this characteristic was not included in the final regression models. This probably reflects the fact that more than half of EDSIG members are not classified as eating disorder specialists: EDSIG members included psychiatrists who were interested, but not necessarily highly experienced, in the treatment of eating disorders. Our conclusion is that it is experience of treating eating disorders, rather than simply an interest in such disorders, that differentiated responses as measured by the factor scores.

**Table 6:** Multiple linear regression analysis results.

		Factor score, mean (SD)					
Respondent Category	N	**Factor 1**		**Factor 2**		**Factor 3**	

**Gender**							

Male	357	7.63	(1.07)	6.40	(1.33)	**5.34**	**(1.57)**

Female	328	7.76	(0.89)	6.48	(1.24)	**5.06**	**(1.41)**

p-value		0.09		0.72		**0.01**	

**Duration of practice**							

More than 10 years	440	7.64	(1.04)	6.48	(1.34)	5.13	(1.50)

Up to 10 years	245	7.79	(0.89)	6.36	(1.20)	5.34	(1.50)

p-value		0.09		0.34		0.07	

**Child and adolescent psychiatrist**							

Child and adolescent psychiatrist	209	7.63	(0.92)	**6.71**	**(1.23)**	5.03	(1.43)

Non-child and adolescent psychiatrist	477	7.72	(1.01)	**6.32**	**(1.30)**	5.27	(1.52)

p-value		0.16		**< 0.001**		0.12	

**Eating disorder specialist**							

Eating disorder	108	**7.93**	**(0.84)**	6.49	(1.50)	**4.85**	**(1.63)**

Non-eating disorder	578	**7.65**	**(1.01)**	6.42	(1.25)	**5.27**	**(1.47)**

p-value		**0.01**		0.36		**0.003**	

**EDSIG membership**							

Member	245	7.74	(0.98)	6.36	(1.34)	5.21	(1.46)

Non-member	441	7.67	(1.00)	6.47	(1.26)	5.20	(1.52)

p-value		0.51		0.14		0.20	

Factor scale scores (on a scale of 0 to 10) by respondent category and the p-value after adjusting for all other respondent categories in a multiple linear regression model. Significant differences are indicated in bold.

Eating disorder specialists scored significantly higher than non-eating disorder specialists on Factor 1 (t = 2.99, d.f. = 160, p = 0.003). They also scored significantly lower than non-eating disorder specialists on Factor 3 (t = -2.49, d.f. = 139, p = 0.014). Child and adolescent psychiatrists scored more highly than non-child and adolescent psychiatrists on Factor 2 (t = 3.623, d.f. = 374, p < 0.001). Men scored more highly than women on Factor 3 (t = 2.46, d.f. = 662, p = 0.014). All these associations remained after adjustment for the other respondent characteristics.

For information, every item on the questionnaire and the distribution of responses items are provided [see Additional file 1].

## Discussion

This article reports on findings of a questionnaire study of psychiatrists' attitudes to anorexia nervosa and the use of compulsory treatment.

### Limitations of the study

There are several limitations to this study. The response rate, though not unusual for response rates of surveys amongst doctors, was not high. Furthermore the child and adolescent psychiatrists and general psychiatrists were sampled only from the South East Region of England, and there were a large number of names removed from the general adult psychiatrists' lists released for research by the Royal College. Caution is therefore needed in trying to generalise from the findings of this survey to the views of senior psychiatrists in England or the United Kingdom in general. Despite these limitations the results provide the most comprehensive evidence we have of psychiatrists' attitudes to anorexia nervosa.

### Summary of the main findings and possible explanations

The respondents in this study showed strong support and consensus for the use of the Mental Health Act, particularly to protect the health and welfare of patients. Most psychiatrists responding to the survey supported the concept of protection from harm for patients with mental disorders, including anorexia nervosa. In contrast, there was more variation in the range of views amongst psychiatrists seen in their responses concerning underlying beliefs about the nature of anorexia nervosa.

Patients with 'mild' anorexia nervosa were generally seen as having significantly more control over behaviour and decision-making ability than those with 'severe' anorexia nervosa. Eating disorder experts were less likely than other psychiatrists to think that patients with 'mild' anorexia nervosa are choosing to engage in weight loss or able to control their weight loss behaviours. Eating disorder specialists may therefore be less likely attribute responsibility for weight loss behaviours to patients who are not yet severely ill. This may have implications for how patients with anorexia nervosa are treated. For example, it may be possible that given their different attitudes, eating disorder experts may be more prepared to act in order to protect the health of their patients who are not yet severely ill from the disorder. This is clinically relevant as patients with relatively mild anorexia nervosa may nevertheless be at some risk to themselves and measures to restrict their freedoms and supervise their behaviours may be considered at an early stage, for example when they are rapidly losing weight. This variation in attitudes may therefore lead to inconsistency between types of psychiatrists in whether compulsory treatment is employed in cases of relatively mild anorexia nervosa when patients are at risk.

On exploratory factor analysis, there were three factors which emerged as contributing a quarter of the variance in item responses. A broadly coherent set of attitudes was found which clustered around three themes of: approval of the use of the Mental Health Act for protection of patients from harm; the primacy of the consideration of best interests of patients with anorexia nervosa; and the preservation of autonomy in anorexia nervosa, as seen in the ability to make choices, retain control and make decisions about treatment.

The variation in attitudes to the nature of anorexia nervosa and its impact upon decision-making was explained to some extent by the type of respondent. Psychiatrists who specialise in treating eating disorders were more likely to give responses suggesting stronger support for the use of mental health legislation to protect patients from harm, and less support for the view of preservation of autonomy in anorexia nervosa. It appears, therefore, that eating disorder specialists in particular, may be more inclined to protect the health and welfare of patients with anorexia nervosa than other psychiatrists: that is, they have lower support for the idea that patients with anorexia nervosa have autonomy (with or without strong support for the use of mental health legislation).

This is consistent with three possible hypotheses. First, that those who choose clinical work involving eating disorder patients may be generally more inclined than other psychiatrists to emphasise protection of patient health when patients may be at risk to themselves. This is relatively unlikely as the treatment of eating disorders tends to involve protracted attempts to engage patients and motivate them to accept treatment. Second, that there is a more protective attitude amongst those involved significantly in eating disorders with regard to prevention of harm in anorexia nervosa – an attitude that has spread and stabilised in the group through shared discussions. This is also relatively unlikely as the use of compulsory treatment in eating disorders is contentious. Third, that eating disorder specialists are particularly aware of the subtle difficulties that patients with anorexia may have in making treatment decisions, and are more inclined as a result to emphasise protection of patients from harm.

Child and adolescent psychiatrists are more likely to give responses suggesting support for the primacy of best interests. This is consistent with the ethos of the Child and Adolescent Psychiatry subspecialty as a whole, which, we expect, gives relatively greater weight to best interests compared with respecting patient choice than does the ethos of Adult Psychiatry.

### Practical and ethical implications of the results

These results may have practical implications for the treatment of patients with anorexia nervosa.

First, the rationale for use of the Mental Health Act (and other legal means of compulsion such as the use of parental consent) for most of the respondents appears to be the protection of patient health and welfare, rather than patients' loss of competence to make treatment decisions. This suggests that the current risk-based mental health legislation may be more consistent with psychiatrists' attitudes than the capacity-based mental health legislation proposed by some [27].

Second, there is considerable variation in attitudes amongst different types of psychiatrists about the nature of anorexia nervosa as well as its impact on decision-making. Given that there is limited and uneven provision of specialist eating disorder treatment across the United Kingdom, the variation of these attitudes between types and levels of eating disorder experience of psychiatrists may provide cause for concern, as patients attending general adult, child and adolescent and specialist eating disorder care settings may be treated differently as a result of the type of psychiatrist they see. On the positive side, given the lack of conclusive evidence about the efficacy (or deleterious effect) of compulsory treatment, the variation between psychiatrists may have the benefit of allowing patients to find a psychiatrist who has the right 'fit' of treatment approach to their particular needs.

Over the last decades there has been increasing emphasis on respecting patient autonomy. The Mental Capacity Act (2005) emphasises this principle as do key medical guidelines [28-30]. The results from this study suggest that in the setting of anorexia nervosa, psychiatrists take, in general, a rather protective approach which includes supporting compulsion when patients are at risk, and this is more marked in those who specialise in the treatment of patients with eating disorders. Such an approach however is not necessarily out of step with the general move towards respect for patient autonomy. This is because the issue of whether patients with anorexia nervosa who refuse treatment are exercising their autonomy is a contentious one. On the whole, those psychiatrists in our sample who emphasised patient protection from harm and the primacy of best interests also viewed the anorexia nervosa as interfering with patients' abilities to make autonomous decisions. This widespread view amongst our sample of experienced psychiatrists might represent an undue level of professional paternalism, or it might indicate that there are problems with the autonomy of patients with anorexia nervosa in the setting of refusing treatment, and that these problems need to be taken seriously in developing policies regarding patient choice and the use of compulsory treatment in mental health services. There is support for this second view from the findings of two separate studies that we have carried out. These findings suggest that patients with anorexia nervosa themselves describe difficulties with autonomously making treatment decisions despite performing well in a formal test of capacity and of being able to express views and to function relatively normally in other aspects of their lives [4,6,7,31].

The question of when it is right to override patient refusal of treatment in the setting of anorexia nervosa remains important and difficult. It involves consideration of the relevance and balancing of different ethical principles as well as of the nature of the disorder itself. The results reported in this article suggest that there is considerable variation even amongst psychiatrists in their attitudes to these issues. Communication and sharing of ideas between different psychiatrists, as well as between psychiatrists, carers and people with anorexia nervosa, are an important way of helping to shape effective practice; there may also be a need for a flexibility of approach according to the different presentations and characteristics of people with anorexia nervosa. Finally, there is a need for further understanding regarding when and how people with anorexia nervosa have difficulty in making treatment decisions, and when and how compulsion can be helpful to them.

## Abbreviations

EDSIG: The Eating Disorder Special Interest Group of the Royal College of Psychiatrists of the United Kingdom and Northern Ireland. Since this research was carried out, the EDSIG has become the Eating Disorders Section of the Royal College of Psychiatrists.

## Competing interests

There are no competing financial or non-financial interests. JT, AS and TH are members of the Royal College of Psychiatrists, and JT and AS are members of its Eating Disorders Special Interest Group. JT's salary and the project costs were funded by the Wellcome Trust through a research fellowship. No other authors received payment for their contributions to the research.

## Authors' contributions

JT developed, conducted and analysed the survey; HD supervised the design and statistical analysis of the questionnaire; RF, TH and AS supervised the development and analysis of the survey; all authors contributed equally to the interpretation of results of the survey. All authors were involved in writing this report.

## Ethics Approval

This project was carried out with multi-centre research ethics approval from the Oxfordshire Research Ethics Committee B, approval reference MREC no. 04/Q1605/21.

## Supplementary Material

Additional file 1 Table 7: Responses to questionnaire items (distribution of responses for each questionnaire item given as valid percentages). Click here for file

## References

[B1] Beauchamp TL, Childress JF (2001). Chapter 3: Respect for autonomy. Principles of Biomedical Ethics.

[B2] Kennedy I, Grubb A (2000). Chapter 5: Consent. Medical Law.

[B3] Grisso T, Appelbaum PS (1998). Chapter 3: Abilities related to competence. Assessing competence to consent to treatment: A guide for physicians and other health professionals.

[B4] Tan J, Hope T, Stewart A (2003). Competence to refuse treatment in anorexia nervosa. International Journal of Law and Psychiatry.

[B5] Tan JO, Hope T, Stewart A (2003). Anorexia nervosa and personal identity: The accounts of patients and their parents. International Journal of Law and Psychiatry.

[B6] Tan JO, Hope T, Stewart A, Fitzpatrick R (2003). Control and compulsory treatment in anorexia nervosa: the views of patients and parents. International Journal of Law and Psychiatry.

[B7] Tan JOA, Hope T, Stewart A, Fitzpatrick R (2006). Competence to make treatment decisions in anorexia nervosa: thinking processes and values. Philosophy, Psychology and Psychiatry.

[B8] Royal College of Psychiatrists (1992). Report to the College Section of General Psychiatry by the Eating Disorders Working Group.

[B9] Royal College of Psychiatrists (2001). CR87. Eating Disorders in the UK: Policies for Service Development and Training. Report from the Eating Disorders Special Interest Group of the Royal College of Psychiatrists.

[B10] Griffiths R, Russell J, Vandereycken W, Beumont PJ (1998). Compulsory treatment of anorexia nervosa patients. Treating eating disorders: ethical, legal and personal issues.

[B11] Ramsay R, Ward A, Treasure J, Russell GFM (1999). Compulsory treatment in anorexia nervosa. British Journal of Psychiatry.

[B12] Watson TL, Bowers WA, Andersen AE (2000). Involuntary treatment of eating disorders. Am J Psychiatry.

[B13] Carney T, Tait D, Saunders D, Touyz S, Beumont P (2003). Institutional options in management of coercion in anorexia treatment: The antipodean experiment?. International Journal of Law and Psychiatry.

[B14] Griffiths RA, Beumont PJ, Russell J, Touyz SW, Moore G (1997). The use of guardianship legislation for anorexia nervosa: a report of 15 cases. Aust N Z J Psychiatry.

[B15] Greenberg D, Mazar J, Brom D, Barer YC (2005). Involuntary outpatient commitment: a naturalistic study of its use and a consumer survey at one community mental health center in Israel. Med Law.

[B16] Whyte B-L, Kaczkowski H (1983). Anorexia nervosa: A study of psychiatrists' and psychologists' opinions and practices. International Journal of Eating Disorders.

[B17] Fleming J, Szmukler GI (1992). Attitudes of medical professionals towards patients with eating disorders. Aust N Z J Psychiatry.

[B18] Jarman M, Smith JA, Walsh S (1997). The psychological battle for control: A qualitative study of health-care professionals' understandings of the treatment of anorexia nervosa. Journal of Community and Applied Social Psychology.

[B19] Alem A, Jacobsson L, Lynoe N, Kohn R, Kullgren G (2002). Attitudes and practices among Ethiopian health care professionals in psychiatry regarding compulsory treatment. Int J Law Psychiatry.

[B20] Alexius B, Berg K, Aberg-Wistedt A (2002). Psychiatrists' perception of psychiatric commitment. International Journal of Law and Psychiatry.

[B21] Romans S, Dawson J, Mullen R, Gibbs A (2004). How mental health clinicians view community treatment orders: a national New Zealand survey. Australian and New Zealand Journal of Psychiatry.

[B22] Roberts C, Peay J, Eastman N (2002). Mental Health Professionals' Attitudes towards Legal Compulsion in England and Wales: Report of a National Survey. International Journal of Forensic Mental Health.

[B23] Greenberg D, Mazar J, Brom D, Barer YC (2005). Involuntary outpatient commitment: a naturalistic study of its use and a consumer survey at one community mental health center in Israel. Med Law.

[B24] Currier GW A survey of New Zealand psychiatrists' clinical experience with the Mental Health (Compulsory Assessment and Treatment) Act of 1992. New Zealand Medical Journal.

[B25] Kullgren G, Jacobsson L, Lynoe N, Kohn R, Levav I (1996). Practices and attitudes among Swedish psychiatrists regarding the ethics of compulsory treatment. Acta Psychiatr Scand.

[B26] Oppenheim AN (1992). Chapter 8: Question wording. Questionnaire Design, Interviewing and Attitude Measurement.

[B27] Dawson J, Szmukler G (2006). Fusion of mental health and incapacity legislation. Br J Psychiatry.

[B28] General Medical Council (1998). Seeking patients' consent: The ethical considerations. General Medical Council.

[B29] Department of Health (2001). Consent – what you have a right to expect: a guide for adults. Department of Health.

[B30] Department of Health (2001). Consent: A guide for children and young people. Department of Health.

[B31] Tan JOA, Stewart A, Fitzpatrick R, Hope T Attitudes of patients with anorexia nervosa to compulsory treatment and coercion.

