# Supplementary material for: Psychiatrists' attitudes towards autonomy, best interests and compulsory treatment in anorexia nervosa: a questionnaire survey
Source: Child Adolesc Psychiatry Ment Health. 2008 Dec 17;2:40. doi: 10.1186/1753-2000-2-40 (PMC2649038; doi:10.1186/1753-2000-2-40)
Supplement: Additional file 1 — Table 7: Responses to questionnaire items (distribution of responses for each questionnaire item given as valid percentages). [file 1753-2000-2-40-S1.doc]

Table 7: Responses to questionnaire items (distribution of responses for each questionnaire item given as valid percentages)

| Part A – Vignette  ‘Mandy is 16 years old, and is being treated in the community for anorexia nervosa. She is reluctant to put on weight as she feels she is too fat. She understands, at least intellectually, that if she continues to lose weight she can put her health and life at risk. Despite outpatient psychological treatment together with dietary advice, she continues to lose weight, and weighs 75% of her expected weight with associated physical symptoms. Medical investigations suggest her situation is medically serious but not yet life-threatening. She is resistant to the doctor’s recommendation to be admitted to hospital. Her parents feel they cannot look after her at home any longer and want her admitted to hospital.’ | | | | | | | |
| --- | --- | --- | --- | --- | --- | --- | --- |
|  | Strongly disagree | Moderately disagree | Slightly disagree | Neither agree nor disagree | Slightly agree | Moderately agree | Strongly agree |
| A1.‘Since Mandy understands the risks, her refusal of treatment should ultimately be respected.’ | 16.6 | 35.8 | 12.0 | 4.1 | 13.2 | 14.2 | 4.0 |
| A2. ‘Since the Mental Health Act permits compulsory treatment in this case, it should be used as she is at substantial risk.’ | 6.6 | 18.4 | 12.8 | 6.3 | 15.9 | 30.4 | 9.6 |
| A3. ‘Since Mandy is young she should be treated in her best interests against her will.’ | 19.6 | 21.5 | 10.8 | 11.0 | 13.7 | 18.0 | 5.4 |
| A4. ‘In the end the parents’ decision should prevail over Mandy’s treatment refusal as she is only 16 years old.’ | 22.8 | 25.3 | 13.4 | 7.2 | 12.8 | 14.0 | 4.4 |
| A5. ‘Although Mandy is intellectually able to understand the risks, the fact that she has anorexia nervosa means that her competence to refuse treatment is almost certainly compromised.’ | 5.7 | 10.3 | 7.2 | 4.6 | 16.2 | 32.8 | 23.2 |
| A6. ‘If Mandy were 13 years old rather than 16 years old, her treatment team should be more willing to override her treatment refusal.’ | 7.8 | 10.7 | 6.3 | 8.7 | 17.9 | 31.9 | 16.6 |
| A7. ‘If Mandy were 25 years old rather than 16 years old, her treatment team should be less willing to override her treatment refusal.’ | 11.7 | 22.3 | 12.5 | 12.6 | 16.6 | 18.2 | 6.0 |
| Part B – Questions on mental disorders in general | Strongly disagree | Moderately disagree | Slightly disagree | Neither agree nor disagree | Slightly agree | Moderately agree | Strongly agree |
| B8. ‘The Mental Health Act should be used more frequently to protect the health and safety of patients.’ | 9.1 | 15.3 | 13.4 | 35.6 | 11.3 | 10.9 | 4.4 |
| B9. ‘The Mental Health Act should not be used when patients are able to make informed treatment decisions, even if they are placing themselves at risk.’ | 6.3 | 18.7 | 16.3 | 7.4 | 15.0 | 24.6 | 11.6 |
| B10. ‘The Mental Health Act should not be used to enforce admission to hospital for mental disorders.’ | 73.7 | 18.4 | 3.2 | 1.2 | 0.9 | 1.3 | 1.3 |
| B11. ‘The Mental Health Act is used too often in the treatment of mental disorders.’ | 13.3 | 23.0 | 14.0 | 31.1 | 12.2 | 4.1 | 2.4 |
| Part C – Questions specifically on anorexia nervosa:  I. Use of the Mental Health Act for anorexia nervosa | Strongly disagree | Moderately disagree | Slightly disagree | Neither agree nor disagree | Slightly agree | Moderately agree | Strongly agree |
| ‘The Mental Health Act should not be used when patients clearly believe that the advantages of anorexia nervosa for them outweigh the disadvantages.’ | 34.2 | 39.4 | 11.5 | 8.3 | 3.2 | 2.8 | 0.6 |
| ‘It is appropriate that the Mental Health Act enables compulsory re-feeding of patients with anorexia nervosa.’ | 1.3 | 3.2 | 1.9 | 3.1 | 11.5 | 42.6 | 36.3 |
| ‘The Mental Health Act is used too often in the treatment of anorexia nervosa.’ | 9.1 | 20.6 | 14.0 | 51.0 | 3.4 | 1.3 | 0.6 |
| ‘The Mental Health Act should not be used to enforce admission to hospital for anorexia nervosa.’ | 42.6 | 38.2 | 10.6 | 4.3 | 1.9 | 1.9 | 0.4 |
| ‘The Mental Health Act should be used more frequently to protect the health and safety of patients with anorexia nervosa.’ | 2.1 | 8.0 | 9.3 | 42.9 | 14.7 | 18.3 | 4.9 |
| Part C – Questions specifically on anorexia nervosa:  II. Treatment decisions in anorexia nervosa | Strongly disagree | Moderately disagree | Slightly disagree | Neither agree nor disagree | Slightly agree | Moderately agree | Strongly agree |
| ‘Parental consent is used too often in the treatment of anorexia nervosa in legal minors.’ | 5.2 | 11.2 | 8.5 | 56.1 | 9.6 | 7.5 | 1.9 |
| ‘Treatment of anorexia nervosa against a patient’s will is justified if it is likely that the patient will recover and have a good outcome after treatment.’ | 3.1 | 5.0 | 5.1 | 9.6 | 16.0 | 32.9 | 28.2 |
| ‘Treatment of anorexia nervosa against a patient’s will is justified if it is likely that the patient will subsequently say he or she is glad that treatment was enforced.’ | 5.9 | 8.2 | 7.1 | 22.1 | 19.6 | 22.4 | 14.9 |
| ‘Treatment refusal by patients is due to the influence of the anorexia nervosa and therefore does not fully reflect their true wishes or personality.’ | 1.5 | 7.5 | 11.3 | 8.0 | 24.3 | 36.8 | 10.6 |
| ‘Palliative care and hospice treatment rather than curative treatment should be an option for adult patients with chronic anorexia nervosa.’ | 16.3 | 18.7 | 9.1 | 17.8 | 19.1 | 13.0 | 5.9 |
| Part C – Questions specifically on anorexia nervosa:  III. Choice and responsibility in anorexia nervosa | Strongly disagree | Moderately disagree | Slightly disagree | Neither agree nor disagree | Slightly agree | Moderately agree | Strongly agree |
| ‘Patients with anorexia nervosa choose to engage in weight loss behaviours – mild anorexia | 3.7 | 11.9 | 8.2 | 7.5 | 25.2 | 32.1 | 11.3 |
| ‘Patients with anorexia nervosa choose to engage in weight loss behaviours – severe anorexia nervosa’ | 20.2 | 28.4 | 9.9 | 6.9 | 11.4 | 13.0 | 10.2 |
| ‘Patients with anorexia nervosa are able to control their own dieting, exercise and purging behaviours – mild anorexia nervosa’ | 5.6 | 17.6 | 15.1 | 5.9 | 29.8 | 21.7 | 4.3 |
| ‘Patients with anorexia nervosa are able to control their own dieting, exercise and purging behaviours – severe anorexia nervosa’ | 34.1 | 35.7 | 9.4 | 5.5 | 8.6 | 3.7 | 3.1 |
| ‘Patients with anorexia nervosa want help even when they are refusing it – mild anorexia nervosa’ | 5.9 | 14.4 | 16.3 | 26.2 | 18.2 | 16.9 | 2.1 |
| ‘Patients with anorexia nervosa want help even when they are refusing it – severe anorexia nervosa’ | 9.8 | 16.7 | 10.2 | 27.4 | 14.2 | 17.8 | 4.0 |
| ‘Patients with anorexia nervosa are generally able to reason properly about treatment – mild anorexia nervosa’ | 5.2 | 22.0 | 23.3 | 10.0 | 21.5 | 15.8 | 2.2 |
| ‘Patients with anorexia nervosa are generally able to reason properly about treatment – severe anorexia nervosa’ | 40.4 | 35.5 | 10.8 | 5.0 | 4.7 | 3.1 | 0.4 |
| ‘Patients with anorexia nervosa have difficulties other than problems with reasoning that make it hard for them to make treatment decisions – mild anorexia nervosa’ | 1.2 | 4.0 | 4.0 | 12.5 | 29.6 | 35.8 | 12.8 |
| ‘Patients with anorexia nervosa have difficulties other than problems with reasoning that make it hard for them to make treatment decisions – severe anorexia nervosa’ | 1.5 | 2.4 | 1.6 | 8.0 | 11.6 | 39.3 | 35.7 |
| Part C - Questions specifically on anorexia nervosa:  IV. The use of the Mental Health Act in anorexia nervosa  ‘Imagine that you are treating a 19-year old female patient who has anorexia nervosa. She is not able to put on weight in the outpatient treatment setting but is refusing day or inpatient treatment. Each statement below is your clinical judgement of her current situation. Please decide the relative importance of each factor below with respect to the decision your clinical team should make about whether or not to place this patient on a Mental Health Act Section 3.’ | | | | | | | |
|  | Not important Very important | | | | | | |
| Importance Score | 1 | 2 | 3 | 4 | 5 | 6 | 7 |
| ‘The patient’s physical health is at risk.’ | 0.1 | 0.9 | 1.0 | 2.7 | 19.0 | 37.0 | 39.3 |
| ‘The patient would die if not given treatment.’ | 0.1 | 0.4 | 0.4 | 1.2 | 2.6 | 14.5 | 80.6 |
| ‘The patient is unable to recognise what is in her own best interests.’ | 1.2 | 2.8 | 3.8 | 11.9 | 23.4 | 31.2 | 25.7 |
| ‘The patient is not making choices consistent with her pre-morbid personality or wishes.’ | 3.1 | 5.1 | 8.4 | 14.0 | 27.2 | 22.9 | 19.4 |
| ‘The patient’s family is unable to support her in the treatment.’ | 6.5 | 12.8 | 14.0 | 21.3 | 22.8 | 15.0 | 7.8 |
| ‘The patient’s family is keen to support use of compulsory treatment.’ | 11.3 | 18.4 | 17.4 | 22.9 | 19.4 | 7.4 | 3.2 |
| ‘The patient’s physical health is at risk.’ | 0.1 | 0.9 | 1.0 | 2.7 | 19.0 | 37.0 | 39.3 |
